# HIV infection and ART use are associated with altered plasma clot characteristics in Black South Africans

**DOI:** 10.1371/journal.pone.0305826

**Published:** 2024-06-25

**Authors:** Shams Bakali, Zelda de Lange-Loots, Anine Jordaan, Marlien Pieters

**Affiliations:** 1 Faculty of Health Sciences, Centre of Excellence for Nutrition (CEN), North-West University, Potchefstroom Campus, Potchefstroom, South Africa; 2 Faculty of Health Sciences, SAMRC Extramural Unit for Hypertension and Cardiovascular Disease, North-West University, Potchefstroom, South Africa; 3 Laboratory for Electron Microscopy, Chemical Resource Beneficiation (CRB), North-West University, Potchefstroom Campus, Potchefstroom, South Africa; Colorado State University, UNITED STATES

## Abstract

**Background:**

Human immunodeficiency virus (HIV) and antiretroviral treatment (ART) are both associated with hypercoagulability. Altered clot properties could be a potential mechanism thereof. We aimed to investigate the association of HIV and ART, with fibrinogen and plasma clot properties in a group of Black South Africans.

**Methods:**

At baseline, 151 newly diagnosed people living with HIV (PLWH) and 176 controls were recruited. Some PLWH subsequently commenced with ARTs (n = 70) while others remained ART-naïve (n = 81). Fibrinogen and clot properties (turbidity assay) were investigated from baseline to 5-year follow-up. A sub-group of 21 women (n = 10 ART-treated; n = 11 ART-naïve) with HIV was systematically selected and matched with 12 controls, and additional clot properties (rheometry, permeability and fibre diameter) were investigated.

**Results:**

Fibrinogen was lower in the HIV groups compared to the controls, while % γ‘ fibrinogen was higher. PLWH had shorter lag times and lower maximum absorbance than the controls (p<0.05). Their CLTs on the other hand were longer. Most variables increased over time in all groups, but differences in the degree of change over time was observed for lag time (p = 0.024) and permeability (p = 0.03). Participants who commenced with ART had a tendency of delayed clot formation (p = 0.08) and increased clot permeability (p = 0.005).

**Conclusion:**

PLWH had lower total fibrinogen concentration and formed less dense clots. They also formed clots that were more difficult to lyse, which likely not resulted from altered clot properties. ART use (NNRTI’s) had a moderately protective effect, delaying clot formation, and increasing clot permeability.

## Introduction

Human immunodeficiency virus (HIV) infection remains a persistent health burden amidst the era of antiretroviral therapy (ART), with over 39 million people infected globally [1,2]. Infection is highly prevalent in sub-Saharan African countries with South Africa listed among countries with the highest prevalence [3,4]. It is estimated that 66% of people living with HIV (PLWH) live in this region [2]. HIV progression is known to be associated with heightened forms of co-morbidities that are largely ascribed to compromised immunity [5,6].

The introduction and advancement of ART have significantly reduced HIV-related complications, increased survival, and improved quality of life among PLWH [7]. The global community persists in employing strategies to improve ART, expand its coverage and enhance compliance among users with a view to improving health and reducing new infection rates [8,9]. However, evidence has emerged that demonstrates cardiovascular disease (CVD) to be a growing concern and a leading cause of morbidity and mortality among PLWH on ART [10–12]. It is reported that ART use is associated with increased inflammation, dyslipidaemias, endothelial lining injuries as well as altered coagulation [13–15].

Not only ART but also HIV itself is known to be associated with hypercoagulability, evident in the reported imbalances of procoagulant, anticoagulant and fibrinolytic factors among PLWH, increasing thrombotic risk [16–18]. Altered clot structure is a known contributing factor in thrombotic-related cardiovascular complications [19], as clots with tightly packed, dense fibrin networks tend to be more resistant to lysis and contributing to thromboembolic events [19,20]. This may result in adverse complications such as stroke and myocardial infarction, which are also evident in PLWH [12,14,17].

Whether HIV and/or ART may contribute to altered clot properties and the pathogenic mechanisms through which this might be affected, is not yet fully understood. Only a limited number of studies to date, have reported on the association of HIV infection as well as some combinations of ART with clot properties [16,21], fibrinogen levels and other variables that determine or predict the presence or formation of thrombotic clots, with contradicting results and no prospective evidence available [16,22–34]. Therefore, this study investigated the cross-sectional as well as the prospective association of HIV status and ART use, with fibrinogen concentration and plasma clot properties in a group of Black South Africans.

## Materials and methods

### Study population and design

**Fig 1:**
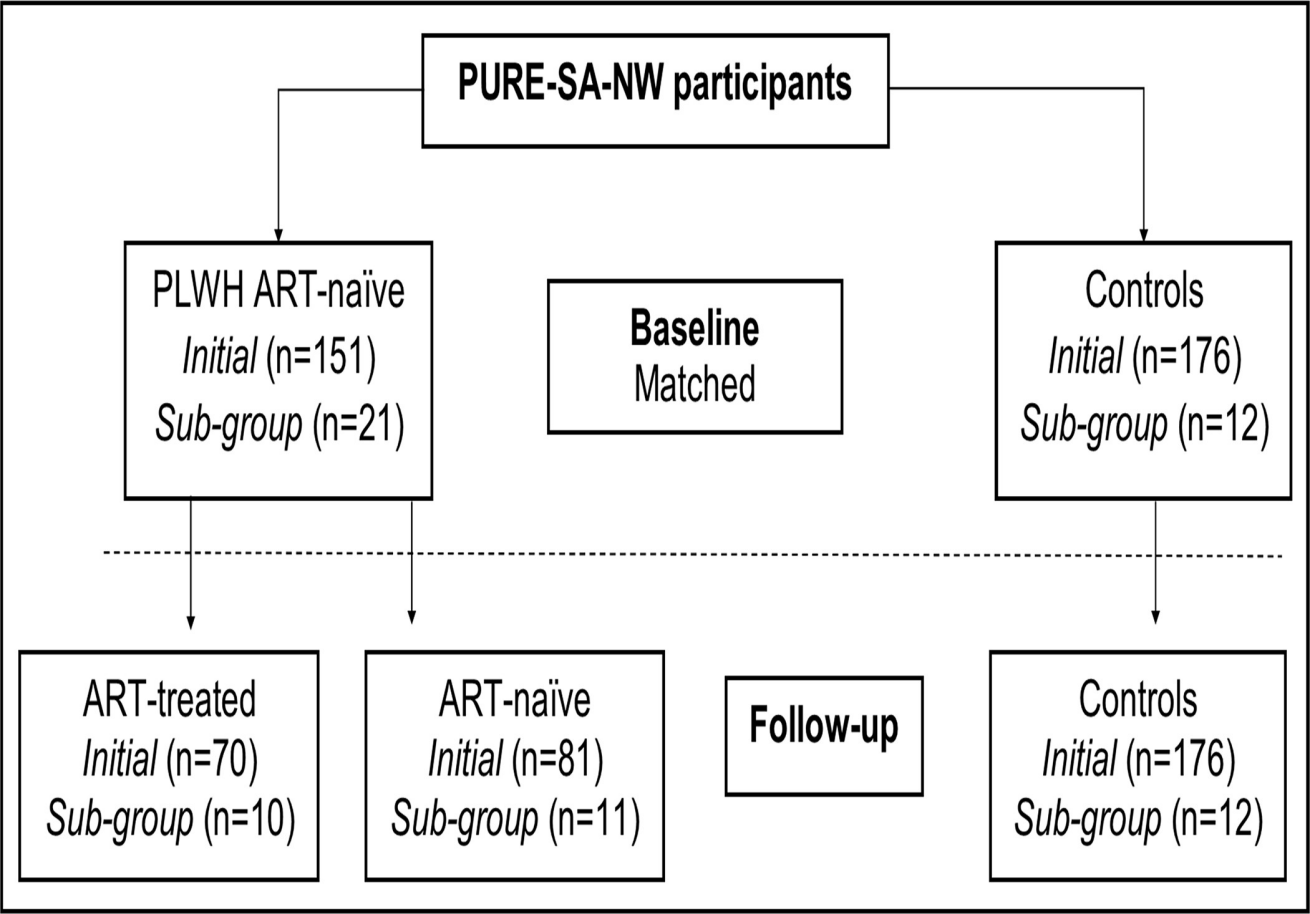
Overview of the study design and participants included from baseline to 5-year follow-up.

This prospective study investigated a group of self-identified Black South Africans, 30 years and older, who participated in the South African North West Province arm of the international Prospective Urban and Rural Epidemiology study [35] over five years (2005–2010) (PURE-SA-NW). Any self-reported prior cardiovascular event, acute illness, pregnancy, or lactation was considered basis for exclusion. Prior to data collection, tympanic membrane temperatures were measured, to exclude individuals with acute illnesses. At baseline, a number of participants (Fig 1) were newly diagnosed as PLWH, and some subsequently commenced ART, under the government ART program, while others did not, as treatment was based on CD4 count at the time (CD4 cell count of ≤200 cells/mm3). Treatment consisted of a combination of ARTs that comprised of two nucleoside reverse transcriptase inhibitors (NRTIs) (Stavudine and Lamivudine), and one non-nucleoside reverse transcriptase inhibitor (NNRTIs) (Efavirenz or Nevirapine).

At baseline, PLWH were systematically matched to HIV-free controls, based on sex, age, and body mass index (BMI), resulting in 151 newly diagnosed, ART-naïve PLWH and 176 matched controls. Of the PLWH, 70 commenced with ART after diagnosis and 81 were still ART-naïve at 5-year follow-up (Fig 1). From these groups, a sub-sample of 12 controls, 10 PLWH that commenced ART and 11 that remained ART-naïve at follow-up were systematically selected for additional clot property analyses. The sub-group included women only considering that this is the sex most affected by HIV in South Africa [36]. The selected women living with HIV were systematically matched to HIV-free women by considering their age, BMI, and oral contraceptive use. Only those with similar oral contraceptive use at both time points were included to exclude the potential confounding effect of change in oral contraceptive use over the five years. Participants with diabetes were also not included. For the larger group, clot properties were determined using a turbidity assay, whilst for the sub-group, clot properties were characterised in more detail by also measuring fibre diameter, permeability, and viscoelasticity. All participants gave written informed consent, and the study was conducted following the revised version (2013) of the Declaration of Helsinki 1975.

### Data collection

Demographic and self-reported information was collected as previously reported [37]. Brachial artery blood pressure (BP) was measured with an Omron HEM-757 automatic digital blood pressure monitor (Omron Healthcare, Kyoto, Japan). Anthropometric measures were performed in accordance with the International Society for the Advancement of Kinanthropometry guidelines [38].

Fasting blood samples were collected by registered nurses. For all haemostatic variables, samples were collected in sodium citrate tubes, and in anticoagulant free tubes for lipids and inflammatory markers. Samples for plasma glucose analysis were collected in sodium fluoride tubes. Samples were centrifuged at 2 000 x g for 15 minutes and stored at -80°C until analysis.

Plasminogen activator inhibitor-1 activity (PAI-1_act_) was quantified using an indirect enzymatic technique (Spectrolyze PAI-1, Trinity Biotech, Bray, Ireland). High-sensitivity C-reactive protein (CRP), total cholesterol (TC), high-density lipoprotein cholesterol (HDL-C), triglycerides (TG), and gamma-glutamyl transferase (GGT) were determined using the Konelab20iTM auto-analyser (Thermo Fisher Scientific Oy, Vantaa, Finland) in 2005 and the Cobas Integra 400 (Roche Clinical System, Roche Diagnostics, Indianapolis) enzymatic colorimetric method in 2010. Glycated haemoglobin (HbA1c) was measured using the D-10 Haemoglobin testing system (Bio-Rad, California, USA). Estimated glomerular filtration rate (eGFR) was calculated with the Chronic Kidney Disease Epidemiology (CKD-EPI) formula and low-density lipoprotein cholesterol (LDL-C) using the Friedewald formula [39]. Soluble urokinase-type plasminogen activator receptor (SuPAR) was measured using a suPARnostic® ELISA kit (ViroGates, Copenhagen, Denmark), whilst intercellular adhesion molecule (ICAM) and vascular cell adhesion molecule/protein (VCAM) were measured by Human sICAM-1 and Human sVCAM-1 sandwich ELISA assays, respectively (IBL international, Hamburg, Germany).

Voluntary human HIV testing was performed with a first response rapid HIV card (PMC Medical, India) and, if positive, confirmed with a further test (Pareeshak card test, BHAT Bio-tech, India). CD4 count was determined using flow cytometry (Beckman Coulter Epics^®^ XL^TM^, Fullerton, USA).

Baseline total fibrinogen concentration was determined by a modified Clauss method with a Multifibrin U-test, BCS analyser (Dade Behring, Deerfield, IL, USA), and an ACL-200 (Automated Coagulation Laboratory analyser from Instrumentation Laboratories, Milan, Italy) at follow-up. Baseline samples (n = 140) were reanalysed together with the follow-up samples to exclude the potential of batch differences that may have resulted from the use of the different analysers. An enzyme linked immunosorbent assay (ELISA) was used to determine fibrinogen γ‘ concentration [40], which is reported here as relative concentration (%) of total fibrinogen concentration.

#### Turbidity assay

Clot properties were determined using a modified global fibrinolytic potential turbidity assay as previously described [41]. Briefly, a tissue factor (Dade Innovin, Siemens Healthcare Diagnostics Inc. Marburg, Germany) and calcium chloride (CaCl_2_) (Merck, Darmstadt, Germany) mixture was used to trigger coagulation, and tissue plasminogen activator (Actilyse, Boehringer Ingelheim, Ingelheim, Germany) was used to initiate clot lysis. Lag time, slope, maximum absorbance and clot lysis time (CLT) were calculated. The same control plasma was used at both time points. The 2005 data were normalised according to the 2010 control values to ensure potential assay drift did not influence the results.

#### Permeability

Clot permeability (K_s_) was measured as described previously by Pieters *et al*., [42]. Plasma samples were clotted by adding human α-thrombin (Merck, Darmstadt, Germany) and CaCl_2_. Buffer was permeated through, and K_s_ calculated.

#### Scanning electron microscopy (SEM) and fibre diameter measurement

Samples from permeability measurements were prepared for SEM imaging by washing with cacodylate buffer and fixed in 2% glutaraldehyde (Merck, Darmstadt, Germany), followed by ethanol dehydration in successive dose increases and chemically dried with hexamethyldisilazane (Merck, Darmstadt, Germany). Clots were coated with carbon, then sputter coated with gold-palladium in preparation for viewing and photographing with an FEI Corporation Quanta 200ESEM (Hillsboro, Oregon, USA). The diameters of 100 systematically selected fibres, in each of five micrographs per individual, were measured using ImageJ (v 1.48, NIH, Bethesda, Maryland, USA).

#### Rheometry

Plasma clots were prepared with human α-thrombin and CaCl_2_.Viscoelastic properties were determined by performing oscillatory shear measurements at 37°C with an ARES-G2 Rheometer (TA Instruments, New Castle, Delaware, USA) as previously described [43]. Storage modulus (G’) and loss modulus (G”), i.e., elastic, and viscous properties, respectively, were measured. The coefficient of variation for all methods was <10%.

### Statistical analysis

Data was analysed using IBM-SPSS software (version 26), and Type-I error was set at 5% (α = 0.05). Continuous data was described using means and standard deviation or median and interquartile range according to variable skewness. Cross-sectional comparisons were performed using consecutive General Linear Regression (GLM) models to determine the independent effect of HIV and ART on fibrinogen and plasma clot properties. Model 1 adjusted for covariates that differed significantly between PLWH and controls, and at the same time correlated with fibrin clot properties (baseline: age, sex, BMI, SBP, HDL-C, GFR, GGT, CRP and ICAM; follow-up: age, sex, SBP, GGT, HbA1c, SuPAR, VCAM, PAI-1_act_, HDL-C and LDL-C). Model 2 additionally adjusted for fibrinogen concentration when clot properties were the outcome measures. Model assumptions of linearity and normality were examined by visual inspection of scatter plots (independent variables against regression residuals) and histograms (using standardised residuals), respectively. Bootstrapping was performed (using 1 000 samples) if the original GLM did not meet homoscedasticity or normality requirements.

Changes over time, within groups were determined with paired t-tests. Mixed linear models were used to determine the differences in the degree of change over time in fibrinogen and plasma clot properties between groups. The interaction p-value was used to interpret the changes over time between the groups. These models were adjusted for covariates that demonstrated differences in changes between the three groups (age, sex, WC, GGT, HbA1c, SuPAR, VCAM, PAI-1_act_, HDL-C and LDL-C). For the sub-sample, a Kruskal-Wallis analysis of variance (ANOVA) was used for cross-sectional comparison as the sample size was too small to allow for adjustment of covariates. Wilcoxon matched pairs test was used for prospective analysis and mixed linear models were used to determine the differences in the degree of change over time between the groups (unadjusted and adjusted for fibrinogen concentration).

## Results

### Descriptive characteristics

Descriptive characteristics of the study participants at baseline are presented in Table 1. Of the PLWH and controls, 70.2% and 60.8%, respectively, were women. PLWH had similar BMI and WC but lower lipid levels than the controls. The controls were slightly older than the PLWH with a median age of 44 years (40–50.5) compared to 42 years (38–48).

**Table 1:** Descriptive characteristics of the study population at baseline.

**Variable**	**PLWH (n = 151)**	**Controls (n = 176)**
Age (years)	42.0 (38.0–48.0)	44.0 (40.0–50.5)
Women n (%)	106 (70.2)	107 (60.8)
Smokers n (%)	96 (63.6)	107 (60.8)
Alcohol consumers n (%)	75 (49.7)	82 (46.6)
WC (cm)	75.0 [9.71]	76.7 [10.3]
BMI (kg/m^2^)	21.2 (18.7–24.6)	21.5 (19.3–25.9)
SBP (mmHg)	122 [17.8]	129 [22.3]
DBP (mmHg)	83.6 [12.6]	86.4 [14.2]
eGFR (mL/min/1.73m^2^)	121 [39.5]	132 [40.4]
GGT (U/L)	40.6 (26.1–71.7)	46.6 (30.6–87)
Glucose (mmol/L)	4.66 [0.85]	4.91 [0.85]
**Variable**	**PLWH (n = 151)**	**Controls (n = 176)**
HbA1c (%)	5.50 [0.44]	5.53 [0.51]
CRP (mg/L)	2.54 (0.69–8.43)	2.21 (0.71–6.5)
SuPAR (ng/ml)	3.69 (2.93–4.64)	3.29 (2.74–4.07)
ICAM (ng/ml)	564 (335,844)	432 (268–720)
VCAM (ng/ml)	917 (511–1644)	496 (314–898)
PAI-1_act_ (U/ml)	3.93 (1.40–7.08)	4.29 (1.25–7.72)
TC (mmol/L)	4.43 [1.19]	5.13 [1.34]
HDL-C (mmol/L)	1.26 [0.53]	1.74 [0.74]
LDL-C (mmol/L)	2.61 [0.90]	2.89 [1.14]
TG (mmol/L)	1.08 (0.81–1.38)	1.0 (0.76–1.38)
Total fibrinogen (g/L)	3.03 [1.71]	3.47 [1.98]
γ‘ fibrinogen (%)	14.3 [7.39]	12.0 [8.18]
Lag time (min)	4.61 [2.06]	4.99 [2.01]
Slope (au/s)	6.18 [4.48]	7.08 [4.18]
Max absorbance (Δau)	0.37 [0.13]	0.44 [0.15]
CLT (min)	58.8 [9.57]	54.7 [10.8]

Normally distributed data presented as mean [standard deviation], and not normally distributed data as median (interquartile range).

### The association of HIV and ART with fibrinogen and plasma clot characteristics

#### Cross-sectional comparisons

Table 2 presents the cross-sectional and prospective differences in fibrinogen concentration and plasma clot characteristics for the initial group. At baseline, for all the outcome variables, the two HIV groups differed from the control group in the same direction, although the differences were not always statistically significant for both groups. There were no significant differences between the two PLWH groups at baseline. Fibrinogen concentration was lower in the HIV groups (PLWH ART-naïve p = 0.008) compared to the controls, while % γ‘ fibrinogen was higher than the control (p<0.05 for both PLWH groups). The PLWH also had a shorter time to clot formation (lag time) (PLWH ART-treated at follow-up p = 0.01) and formed less dense clots (lower maximum absorbance) than the controls (p<0.05 for both PLWH groups). Their CLTs on the other hand were longer than those of the controls (PLWH ART-naïve p = 0.002). The results remained after adjusting for covariates and additional adjustment for fibrinogen for clot properties as outcomes (Model 2).

**Table 2:** Comparison of baseline to follow-up data in the ART-treated, ART-naïve and control groups (initial group).

Variables	PLWH ART-treated at follow-up (n = 70)			PLWH ART-naïve (n = 81)			Controls (n = 176)			Interaction p-value
	Baseline^ Mean [SD]	Follow-up Mean [SD]	p-value	Baseline Mean [SD]	Follow-up Mean [SD]	p-value	Baseline Mean [SD]	Follow-up Mean [SD]	p-value	
Total fibrinogen (g/L)	3.04 [1.86]	3.32 [0.88]*	0.22	3.01 [1.59]^a^	3.42 [0.90]^#^	0.03	3.47 [1.98]^a^	3.77 [1.09]*^#^	0.09	0.83
γ‘ fibrinogen (%)	14.3 [7.68]^a^	10.3 [3.76]	0.001	14.3 [7.20] ^b^	10.9 [4.23]	0.001	12.0 [8.18]^ab^	10.1 [3.23]	0.008	0.13
Lag time (min)	4.34 [1.96]^a^	4.88 [0.91]	0.08	4.85 [2.12]	4.88 [0.94]	0.7	4.99 [2.01]^a^	4.69 [0.82]	0.06	0.02
Slope (au/s)	5.93 [4.80]	7.27 [2.72]	0.05	6.38 [4.22]	7.64 [2.66]	0.02	7.08 [4.18]	8.51 [2.53]	<0.001	0.87
Max absorbance (Δau)	0.34 [0.11]^a^	0.48 [0.15]*	<0.0001	0.39 [0.13]^b^	0.51 [0.16]	<0.0001	0.44 [0.15]^ab^	0.59 [0.18]*	<0.0001	0.13
CLT (min)	57.5 [9.72]	64.1 [10.1]*	<0.0001	60.0 [9.34]^a^	62.4 [10.2]^#^	0.08	54.7 [10.8]^a^	60.6 [8.76]*^#^	<0.0001	0.16

^ = This group was ART-naïve at baseline.

^ab^ = significant differences between groups at baseline (Model 1 p-values for total fibrinogen and γ‘ fibrinogen and Model 2 p-values for the clot properties).

*^#^ = significant differences at follow-up (Model 1 p-values for total fibrinogen and γ‘ fibrinogen and Model 2 p-values for the clot properties).

At follow-up, both the group that received ART (p<0.001) and the ART-naïve group (p = 0.002), had significantly lower fibrinogen concentrations than the controls. However, there was no difference in fibrinogen concentration between the two groups of PLWH. The group that was ART-treated at follow-up also had lower clot density compared to the controls (p = 0.01), but neither of these groups differed from the ART-naïve group. Both PLWH groups had significantly longer CLTs than the control group (ART-treated at follow-up p = 0.01 and ART-naïve group p = 0.05) but did not differ from each other. There were no significant differences in % γ‘ fibrinogen, time to clot formation or rate of clot formation between the three groups at follow-up. Additional adjustment for fibrinogen and covariates did not significantly alter the clot property results.

#### Changes over time

A significant increase in total fibrinogen, from baseline to follow-up, was observed in the ART-naïve group (p = 0.03). Percentage γ‘ fibrinogen decreased significantly in all three groups. Both the rate of clot formation (borderline in the ART-treated group) and clot density increased over time in all three groups. CLT increased significantly in the group that received ART at follow-up and the control group (p<0.0001, both), with no significant change in the ART-naïve group.

**Fig 2:**
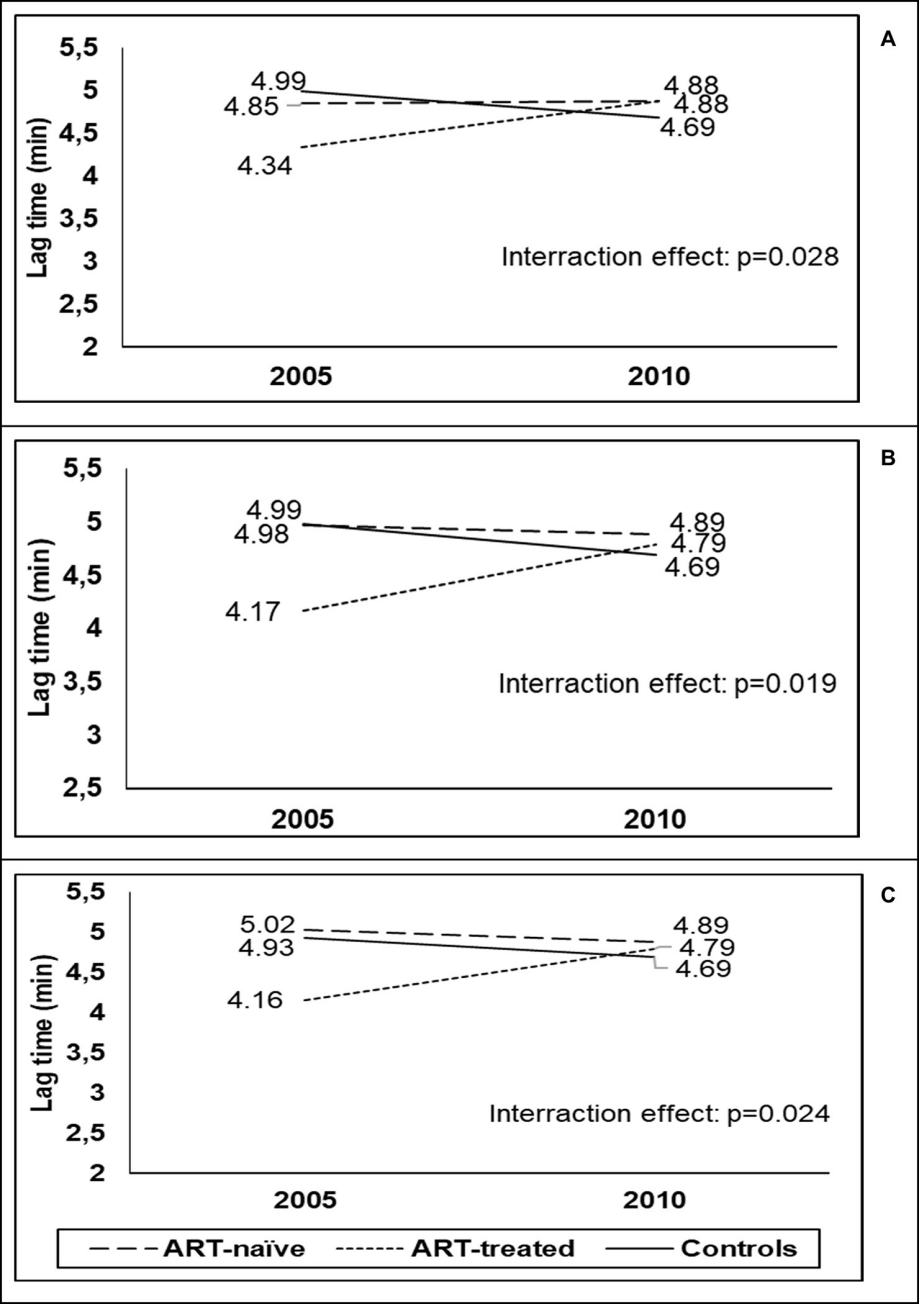
The between-group difference in the degree of change over time for lag time. (A) presents the unadjusted model, (B) is Model 1, which adjusted for covariates that demonstrated differences in changes between the three groups, whilst (C) is for Model 2, which additionally adjusted for fibrinogen concentration. The interaction p-value represents the statistical significance of the differences.

A between-group difference in the degree of change over time was only observed for lag time (p = 0.03) (Fig 2), with a tendency to increase in the ART-treated group (p = 0.08), no change over time in the ART-naïve group and a tendency of decreased lag times in the control group (p = 0.06) (Table 2).

#### Sub-group analyses

There were no statistically significant cross-sectional differences in the measured clot properties between the three groups at either time point. The permeability of the ART-naïve group at follow-up, however, tended to be lower than that of the controls (p = 0.06) (Table 3).

**Table 3:** Comparison of baseline and follow-up data in ART-treated, ART-naïve and control groups (sub-group).

Variables	ART-treated at follow-up (n = 10)			ART-naïve (n = 11)			Control (n = 12)			Interaction p-value
	Baseline	Follow-up	p-value	Baseline	Follow-up	p-value	Baseline	Follow-up	p-value	
Fibre diameter (nm)	116 (104–129)	172 (141–181)	0.005	136 (121–149)	162 (145–175)	0.01	129 (117–144)	175 (143–185)	0.003	0.35
Permeability (cm^2^ x 10^−9^)	3.30 (2.10–5.30)	7.50 (5.40–9.90)	0.005	5.30 (4.20–6.90)	4.10 (3.40–7.30)	0.79	5.10 (3.40–8.20)	7.90 (6.00–9.90)	0.21	0.03
Storage modulus (G’) (Pa)	44.7 (24.0–54.6)	49.9 (24.2–78.2)	0.17	26.1 (11.9–48.7)	56.6 (36.00–100)	0.008	17.3 (15.5–38.6)	40.5 (21.1–66.6)	0.03	0.52
Loss modulus (G”) (Pa)	1.51 (1.09–1.99)	1.66 (1.20–2.79)	0.24	1.26 (0.72–1.93)	2.34 (1.41–4.96)	0.01	1.10 (0.86–1.42)	1.80 (1.19–2.54)	0.03	0.35
Tan Δ (G’/G”)	0.04 (0.04–0.05)	0.03 (0.03–0.05)	0.17	0.05 (0.04–0.06)	0.04 (0.03–0.04)	0.02	0.05 (0.04–0.06)	0.04 (0.03–0.05)	0.14	0.56

Results presented as median (25^th^-75^th^ percentile); Interaction p-value of Model 2 (adjusted for fibrinogen concentration) obtained through linear mixed model analysis.

**Fig 3:**
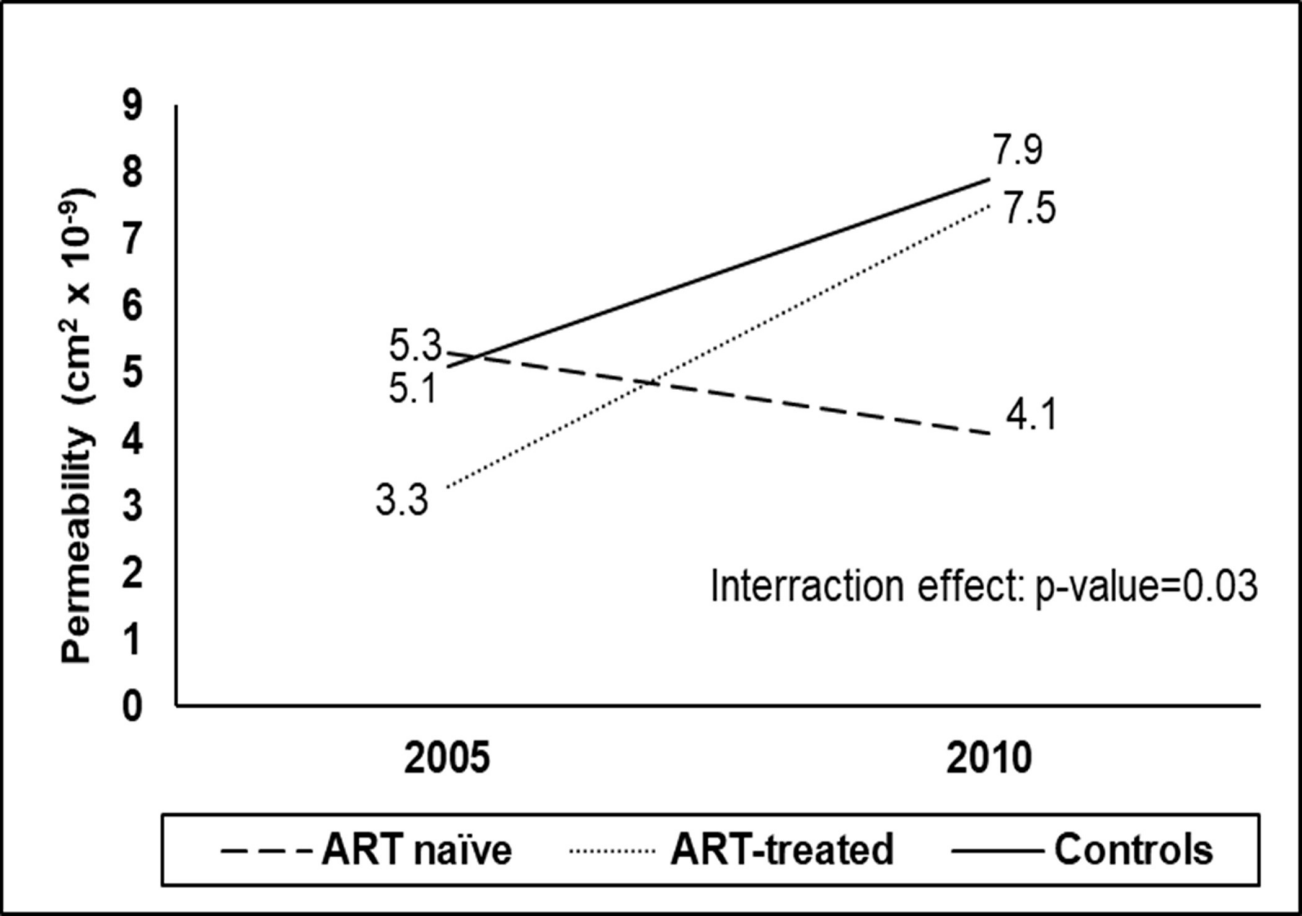
The between-group differences in the degree of change over the 5-year period for permeability in the sub-group. The interaction p-value represents the statistical significance of the differences.

Fibre diameter increased significantly from baseline to follow-up in all three groups. Permeability increased significantly in the group that was ART-treated at follow-up (p = 0.005), while no change was seen in the ART-naïve group with a non-significant increase in the control group. This resulted in a significant difference (p = 0.03) in the degree of change over time between the three groups (Fig 3).

In terms of mechanical properties, both G’ and G” increased significantly in the ART-naïve (p = 0.008 and p = 0.01, respectively) and control (p = 0.03, for both) groups, while no changes were observed in the group that was ART-treated at follow-up. A significant decrease in TanΔ (G’/G”) was observed only in the ART-naïve group, with no significant changes in the other groups.

## Discussion

This study aimed to provide evidence for the potential role altered fibrin clot properties may play in the hypercoagulable phenotype associated with HIV and ART use. PLWH had lower fibrinogen concentration, higher % γ‘ fibrinogen (at baseline), and lower maximum absorbance than controls. Maximum absorbance is indicative of overall density of plasma clots [43], thus indicating the formation of less dense clots in PLWH. CLTs were, however, longer than in controls. Changes over time in the three groups generally did not differ between the groups, with an increase in fibrinogen concentration, fibre diameter, and storage (elastic response or stiffness) and loss (viscous response or inelastic component) modulus, a decrease in % γ‘ fibrinogen, faster rates of lateral aggregation, denser clots and longer CLTs. The only differences between the groups were for lag time and permeability. While time to clot formation tended to become shorter in controls, it remained unchanged in ART-naïve PLWH and tended to increase in PLWH who commenced with ART. Clots of women who commenced with ART also became more permeable over time compared to the ART-naïve and control groups.

The fibrinogen concentration in the PLWH was lower than in the controls even after adjustment for covariates that correlated with fibrinogen and differed between the PLWH and controls. This suggests that the lower fibrinogen concentration likely arose from other (not measured) mechanisms related to HIV. These results concur with a previous publication that reported on a larger sample of the same study population [25]. The study aimed to determine how known polymorphisms within the fibrinogen gene influence the change in fibrinogen over the same 5-year period and also showed the lower fibrinogen concentration in PLWH [25]. The researchers found a higher prevalence of the common allele of the single nucleotide polymorphism (SNP) at rs4220 among PLWH, which was associated with lower fibrinogen concentration, suggesting a genotype-HIV interaction as a possible reason behind the observed results [25]. This SNP is situated in exon 8 of the *FGB* gene. The data from this study contradicts previous findings of studies that have reported higher total fibrinogen concentration in PLWH compared to controls [28,29,31]. However, most of these studies were conducted in ART-treated PLWH, suggesting that their results may have been influenced by the ART treatment, since certain ART regimens, such as protease inhibitors, which were used in those studies, are known to be associated with higher fibrinogen concentration [28,31]. NNRTIs, such as the ARTs used in this study population on the other hand, have also been found to be associated with lower fibrinogen [31].

PLWH formed less dense clots than controls. This suggests that PLWH may form clots with a lower thrombotic risk than healthy controls since increased clot density is generally indicative of a prothrombotic clot phenotype [43,44]. The reason for the lower clot density in the PLWH deserves further investigation. Clot density remained lower also after adjustment for fibrinogen and the measured covariates suggesting additional mechanisms related to HIV likely affect clot structure. Studies have shown for example, that LDL-C can bind to fibrin fibres, increasing clot density and stiffness [41,43,45,46]. In the current study, LDL-C correlated with maximum absorbance and was lower in the PLWH than the control group providing one possible pathway contributing to the observed association.

Contrary to the decreased clot density, CLTs were longer in PLWH than the controls. PLWH also presented with higher % γ‘ fibrinogen compared to the controls. Increased % γ‘ fibrinogen is associated with the formation of clots that are resistant to lysis due to its effects on fibrin clot structure [47,48]. This may have contributed to the longer CLTs observed in PLWH. It is, however, likely that the increased CLT in PLWH may also be related to factors other than clot properties, since adjustment for γ‘ fibrinogen did not fully explain the differences in CLT and PLWH formed less dense clots, which is typically associated with faster lysis rates [44]. CLT is also influenced by a number of other proteins in the coagulation and fibrinolytic pathways such as plasminogen, tissue plasminogen activator, thrombin activatable fibrinolysis inhibitor, PAI-1 and α2-antiplasmin [49,50]. Of these, only PAI-1_act_ was measured in this study, however, adjustment for PAI-1_act_ did not significantly alter the results. Additionally, evidence suggests that inflammatory markers such as CRP, may alter clot formation by interacting with fibrinogen, and can also bind to plasma fibrin clots, consequently influencing CLT, particularly in individuals in a pro-inflammatory state [51–53]. Therefore, elevated levels of inflammatory markers such as CRP, SuPAR, ICAM and VCAM in PLWH, may contribute to the observed prolonged CLT.

Changes in clot properties over the 5-year period were generally similar between PLWH and controls, with clot properties becoming more prothrombotic over time. At follow-up, the PLWH had been living with HIV for five years longer than at baseline (newly diagnosed), therefore, particularly in the ART-naïve group, their risk for HIV-related complications had potentially increased [54]. The CD4 count of the ART group was 20% higher (400 cells/mm^3^) than that of the ART-naïve group (331 cells/mm^3^), suggesting that the weakened immunity among the untreated individuals may result in HIV-related opportunistic infections, as well as cardiovascular complications [55,56]. ART use indeed seemed to have a somewhat protective effect on clot properties of PLWH. Lag time tended to increase in PLWH who commenced with ART. Lag time is the time required for protofibrils to reach sufficient length to allow lateral aggregation [57,58], and hence, increased lag time indicates delayed clot formation. In agreement with our results, Rönsholt *et al*., [21] reported delayed clot formation using thromboelastography (TEG) in Type 1 HIV patients on long-term combination ART compared to healthy controls. Clots that take longer to form tend to have thicker and more loosely packed fibres [59,60]. This finding is supported by the increase in clot permeability observed in PLWH who commenced with ART.

Major strengths of the study are the detailed phenotypic data available for the study population and the prospective design, however, despite the prospective associations found, causality cannot be established. A limitation of the study is the small sample size of the subsample, which may have resulted in a Type-II statistical error. However, the clot properties analysed in the subsample is very time consuming, limiting the number of samples that can be analysed. Since the analyses performed for the sub-sample were done only recently, it is possible that the results may have been affected by the long-term frozen storage. Residual confounding cannot be excluded as a number of coagulation and fibrinolytic proteins related to clot formation and lysis, were not analysed. Our results are limited to Black South Africans and extrapolation to other populations and individuals with different HIV strains should, therefore, be done with caution.

In this Black South African population, HIV was associated with lower fibrinogen concentration and the formation of less dense clots. PLWH, however, also formed clots which were more difficult to lyse. This could be related to altered levels of other variables affected by the HIV status, rather than to altered plasma clot structure. ART use (NNRTI’s) seemed to have a moderately protective effect on plasma clot properties, delaying clot formation, and increasing clot permeability. Future research should focus on identifying mechanisms through which ART use potentially influences clot structure and how clot lysis can be improved in PLWH as a strategy to lower CVD risk.

## Supporting information

S1 File Plasma clot properties for newly diagnosed ART-naïve PLWH and HIV-free controls at baseline. This is a supplementary table that compares plasma clot characteristics of the newly diagnosed PLWH and the controls at baseline, both initial group and sub-group. The data have been presented as mean [standard deviation] for the initial group, and median (25th-75th percentile) for the sub-group. The p-value represents the statistical significance of the differences between the two groups (independent t-test).
(DOCX)
