# Supplementary material for: HIV infection and ART use are associated with altered plasma clot characteristics in Black South Africans
Source: PLoS One. 2024 Jun 25;19(6):e0305826. doi: 10.1371/journal.pone.0305826 (PMC11198788; doi:10.1371/journal.pone.0305826)
Supplement: S1 File — This is a supplementary table that compares plasma clot characteristics of the newly diagnosed PLWH and the controls at baseline, both initial group and sub-group. The data have been presented as mean [standard deviation] for the initial group, and median (25th-75th percentile) for the sub-group. The p-value represents the statistical significance of the differences between the two groups (independent t-test). (DOCX) [file pone.0305826.s001.docx]

**HIV infection and ART use is associated with altered plasma clot characteristics in Black South Africans**

Supplemental Digital Content/supporting information

**Supplemental digital content table 1.** Plasma clot properties for newly diagnosed ART-naïve PLWH and HIV-free controls at baseline

| **Variables** | **PLWH** | **Controls** | **p-value** |
| --- | --- | --- | --- |
|  | *Initial group (n=151)* | *Initial group (176)* |  |
| Total fibrinogen (g/L) | 3.03 [1.71] | 3.47 [1.98] | 0.013 |
| γ’ fibrinogen (%) | 14.3 [7.39] | 12.0 [8.18] | 0.05 |
| Lag time (min) | 4.61 [2.06] | 4.99 [2.01] | 0.246 |
| Slope (au/s) | 6.18 [4.48] | 7.08 [4.18] | 0.212 |
| Maximum absorbance (∆au) | 0.37 [0.13] | 0.44 [0.15] | 0.001 |
| CLT (min) | 58.8 [9.57] | 54.7 [10.8] | 0.003 |
|  | *Sub-group (21)* | *Sub-group (n=12)* |  |
| Fibre diameter (nm) | 123 (113-147) | 129 (117-144) | 0.75 |
| Permeability (cm^2^ x 10^-9^) | 4.7 (2.5-5.7) | 5.1 (3.4-8.2) | 0.21 |
| Storage modulus (G’) (Pa) | 34.6 (15.2-51.6) | 17.3 (15.5-38.6) | 0.37 |
| Loss modulus (G”) (Pa) | 1.38 (0.88-1.93) | 1.10 (0.86-1.42) | 0.31 |
| Tan Δ (G’/G”) | 0.04 (0.04-0.05) | 0.05 (0.04-0.06) | 0.27 |
| Complex viscosity (Pa.s) | 6.92 (3.05-10.3) | 3.46 (3.10-7.72) | 0.37 |

Initial group: The data have been presented as mean [standard deviation]

Sub-group: age, BMI, hormonal contraceptive use-matched women; results presented as median (25^th^-75^th^ percentile)
